# The Impact of Carbon Trade on Outsourcing Remanufacturing

**DOI:** 10.3390/ijerph182010804

**Published:** 2021-10-14

**Authors:** Xiqiang Xia, Mengya Li, Biao Li, Hao Wang

**Affiliations:** 1 School of Business, Zhengzhou University, Zhengzhou 450001, China; xqxia@zzu.edu.cn (X.X.); alexlevi@126.com (M.L.); 2 Department of Geography and Planning, University of Toronto, Toronto, ON M5S 2E8, Canada; haow.wang@mail.utoronto.ca

**Keywords:** carbon trade, outsourcing remanufacturing, game model

## Abstract

Outsourcing remanufacturing is an important way to achieve resource recycling, green manufacturing and carbon neutrality goals. To analyze the impact of carbon trade on manufacturing/remanufacturing under outsourcing remanufacturing, this article builds a game model between an original equipment manufacturer (OEM) and a remanufacturer under the carbon trade policy. In the outsourcing remanufacturing model, this article compares the impact of the carbon trade policy on the unit retail price, sales volume, revenue, environmental impact, and consumer surplus of new and remanufactured products. The research mainly draws the following conclusions: (1) Carbon trade increases the prices of both new and remanufactured products and the cost of outsourcing. Only when certain conditions are met can increased carbon trade prices increase revenue. (2) The carbon trade policy helps reduce the adverse impact on the environment, but only when the carbon trade price is greater than a certain threshold can it increase consumer surplus. (3) Consumer preferences and carbon emissions of the unit product affect manufacturers’ profits. Increased consumer preference for remanufactured products and reduced carbon emissions of remanufactured products contribute to increased sales and revenues.

## 1. Introduction

Human activities are accelerating environmental degradation and resource depletion. While renewable energy has grown significantly worldwide, total global carbon emissions have not been effectively controlled [1]. In 2019, total greenhouse gas emissions reached a record high of 59.1 billion tons of carbon dioxide equivalent. Although global energy consumption slowed down in 2020 due to the COVID-19 pandemic, most countries have not yet reached peak carbon emissions as total carbon emissions are still rising. By 2020, the global carbon dioxide concentration had risen from 280 ppm to 412 ppm, causing global average temperatures to be 1.2 degrees Celsius warmer than they were before the industrial revolution. As a common goal of human beings, most countries have committed to achieving carbon neutrality by the middle of this century to promote low carbon sustainable development. Developed countries such as the United Kingdom have fully achieved carbon peaks and many Asian countries are following suit. However, for some other countries, such as China, it is difficult to rely entirely on market forces to peak carbon emissions, which necessitates strong government intervention [2]. Carbon trade, as an important pricing mechanism for greenhouse gas emissions, is an effective policy tool for countries to address climate change [3].

Carbon trade refers to the policy whereby a government grants each company a fixed amount of carbon emission credits, allowing companies to trade the credits as a scarce resource, and companies with surplus carbon emission credits can sell the remaining to manufacturers who exceed the specified carbon emission credits [4]. This policy increases the cost of carbon emissions and forces companies to seek low-carbon production methods to achieve carbon emission reduction. Therefore, the carbon trade policy can influence firms’ production decisions, forcing them to seek new production strategies to maximize their benefits. In 2005, the European Union established a carbon trade system. The EU-ETS trade system is an emission reduction mechanism based on total volume trading. The emission reduction indicators in the Kyoto Protocol are allocated to each member country in the form of quotas. According to the agreement, the carbon emission allowances of the member countries of the EU-ETS system will be gradually reduced in three stages. The carbon emission reduction industry has expanded from heavy industrial industries such as power plants and steel plants to ammonia production companies and other chemical industries that directly emit greenhouse gases. The EU-ETS system has problems such as unfair distribution of carbon emissions and conflicts with other trade mechanisms [5,6,7,8,9]. In 2021, China used the power sector as a breakthrough to establish the world’s largest carbon market. China’s carbon trade system avoids to a certain extent the excess quotas of EU-ETS due to the “grandfather law” and has certain advantages in price control mechanisms. However, there are also problems such as low transaction scale and small number of industries involved. Carbon prices are affected by various factors such as industrial structure, international carbon trade prices, and government price caps. Regarding the allocation of carbon emission allowances, countries adopt a pattern that combines free allowances and paid allowances, with paid allowances as the mainstream. The scope of the market and the range of industries for carbon trade will be expanded in the future, as the carbon trade market matures. The main carbon pricing mechanisms include price-based ones, such as carbon taxes, and quantity-based mechanisms, such as carbon trade. Scholars have compared the two mechanisms [10] and examined a dual-track model that combines both carbon taxes and carbon trade [4]. Studies on carbon trade mainly focus on the design of total allowances [11], the impact of coverage [12], the principle of allowance allocation [12], the flexible control measures [13], and quota withdrawal mechanism [14].

There are three main mechanisms of carbon emission reduction: (1) by output adjustment, using carbon sinks to replace traditional carbon-heavy outputs (e.g., coal) or using capture and storage to reduce generated carbon emissions. (2) By technological change—achieving low-carbon, zero-carbon, and secondary-carbon development through carbon reduction or decarbonization technology. (3) By energy optimization—replacing traditional carbon emission industries with blue carbon such as hydropower and ocean energy. Remanufacturing is one type of energy optimization, which refers to new technology for recycling, processing, and integrated manufacturing of traditional products [15]. It is a typical green manufacturing technology that achieves carbon reduction or carbon-free manufacturing through technological changes. With rapid changes to new products, the number of waste products has increased dramatically, posing great challenges to the sustainability of the economy [16,17]. Remanufacturing helps reduce the pollution of manufacturing activities to the environment, diminish the carbon emission cost of enterprises, and increase the profits of the carbon trade. This further motivates firms to carry out remanufactured production [18,19,20].

At present, OEMs face barriers to remanufactured production. Many OEMs, for instance, lack the skilled staff, required technology, equipment, and sufficient public support for remanufacturing activities. Additionally, the overall profits of remanufactured product are less than those of creating new products [21,22,23]. As a result, outsourcing the remanufacturing process has become a feasible and popular option for OEMs, in a process known as outsourcing remanufacturing. In particular, OEMs are more willing to use outsourcing remanufacturing technologies when consumers perceive a higher value in remanufactured goods [24,25]. In this model, the OEM outsources the remanufacturing operation to a remanufacturing firm by paying a certain outsourcing fee. In turn, the remanufacturing firm makes the remanufactured products at the agreed quantity. Finally, the OEM sells these products for a profit. Combined with its reverse recycling channels and cost and profit factors, OEM’s recycling can be more beneficial in environmental, economic, and social terms [26,27]. To promote a thriving remanufacturing industry, countries adopt a wide range of intervention policies to facilitate the production of low-carbon products by both OEMs and remanufacturers [28]. Specific policies include production support policies such as government subsidies [29,30,31,32,33,34,35,36,37,38], return policies [39,40,41], and carbon emissions intervention policies such as carbon trade [3,4], carbon taxes [42,43,44,45,46] and carbon regulation [47]. Scholars have compared the impacts of different policies [48], including carbon tax and carbon trade [4,49], carbon taxes and government subsidies [50,51,52,53,54], carbon tax and take-back legislation [55], take-back and carbon emission capacity regulations [47], and government subsidies and carbon regulation [22]. Carbon trade mainly affects the supply and demand of high-carbon new products and low-carbon remanufactured product through the administrative means of carbon emission reduction allowances. In this process, remanufactured products compete with new products and thus affect the sales of new products. Therefore, under the carbon trade policy, OEMs need to consider production costs, carbon trade costs, sales revenue of the two types of products, and the carbon trade profits when making production decisions. Under this condition, carbon trade promotes the growth of the remanufacturing industry in both the ordinary market and the green market [3].

Existing studies have analyzed the pricing mechanism of carbon trade on remanufacturing [3,4,11], but have paid less attention to the impact of carbon trade on outsourcing remanufacturing activities and the different levels of costs associated with these activities. This article establishes a game model of new products and remanufactured product under the carbon trade policy. It examines the effects of carbon trade prices on sales volume, unit product price, revenue, environment, and consumer surplus of the supply chain participants. Furthermore, it analyzes how the carbon trade policy impacts OEMs/remanufacturers’ decision-making mechanism. This article mainly answers the following three questions:

1. What are the impacts of outsourcing remanufacturing on manufacturing/ remanufacturing activities? How do an OEM’s decisions to outsource remanufacturing influence the development of remanufacturing?
2. What are the impacts of carbon trade on manufacturing/remanufacturing activities in the context of outsourcing remanufacturing, such as the price, demand, revenue, environment, and consumer surplus of the two products?
3. What are policy implications for promoting the growth of the remanufacturing industry?

This article is organized into five sections. Section 2 introduces the research questions and the game theory model. Section 3 describes the model construction process, explains the main research conclusions, and proposes implications. Section 4 conducts a numerical case in the context of China. Section 5 summarizes the conclusions and future research directions.

## 2. Model Formulation

### 2.1. Problem Description

Outsourcing remanufacturing refers to a remanufacturing pattern by which an OEM pays specialized remanufacturers to produce remanufactured products and then sells them on the market. This article develops a model in which the remanufacturing supply chain is composed of one OEM and one remanufacturer under the carbon trade policy. Lacking the necessary technology, the OEM outsources the remanufacturing operation to the remanufacturer for production. Both the OEM and the remanufacturer seek to maximize their benefits. In the outsourcing model, the remanufacturer decides on the number of waste products generated in the production process to be recycled, which is affected by the outsourcing fee, i.e., the higher the outsourcing fee, the stronger the remanufacturer’s production incentives, and the greater the number of waste products to be recycled. Apart from the outsourcing fee, the OEM also needs to determine the unit retail price of new and remanufactured products. Given the substitute effect between the new and remanufactured products, the carbon trade policy restricts carbon emissions by limiting the market prospects for new products and strengthening the incentives for remanufacturers. OEMs thus need to consider the retail prices of the two products and the outsourcing fee under the carbon trade policy in order to maximize their benefits. 

Taking the carbon trade policy and government preset prices into consideration, this article establishes a game model between an OEM and a remanufacturer. It analyzes the impact of these two factors on both the OEM’s and remanufacturer’s decisions. According to the reverse order of game theory, the remanufacturer decides the number of waste products to be recycled first. The OEM then determines the outsourcing fee and the unit retail price of the two products.

### 2.2. Model Symbol

Table 1 gives the basic definitions of the symbols used in this article.

### 2.3. Model Function

#### 2.3.1. Model Demand Function

According to [21,48], this article adopts a relatively mature demand function. The relationship between the demand and the unit retail price is as follows:

$$p_{n}=1-q_{n}-\delta q_{r},p_{r}=\delta (1-q_{n}-q_{r})$$

#### 2.3.2. Model Recovery Function

According to [24], the number of recycling waste products is $q_{r}=\tau q_{n}$, the cost of recycling waste products is proportional to the number of recycling, that is, the cost of recycling waste products can be expressed as $\frac{k}{2}{\left(\tau q_{n}\right)}^{2}$, where k is the coefficient of recycling waste products. 

### 2.4. Research Hypothesis

**Hypothesis** **1.** *Under the protection of intellectual property rights, OEMs transfer remanufacturing production to remanufacturers through outsourcing remanufacturing, and the OEM pays the outsourcing remanufacturing costs according to the production quantity of the remanufactured products. OEM influences the remanufacturing enthusiasm of remanufacturers by changing the unit outsourcing remanufacturing expenses.*

**Hypothesis** **2.** *New products and remanufactured products produced by enterprises will have different impacts on the environment. Take the remanufactured engine as an example, compared with the unit new engine, the unit remanufactured engine can reduce carbon emissions by 60%. Therefore, OEMs can influence the volume of carbon trade by changing the production volume of new products and remanufactured products, and ultimately change the revenue of OEMs.*

**Hypothesis** **3.** *The carbon trade policy is an important policy that affects the development of the outsourcing remanufacturing industry. There are many policies that affect the remanufacturing industry, such as government subsidy policies, carbon tax policies, and carbon restraint policies. At present, there are few studies on the impact of carbon trade on remanufacturing, especially the impact of carbon trade on outsourcing remanufacturing.*

## 3. Model Analysis

### 3.1. Model Construction


(1) $$\pi_{n}=(p_{n}-c_{n})q_{n}+(p_{r}-w)q_{r}-(e_{n}q_{n}-T)Q\;$$

(2) $$\pi_{r}=(w-c_{r})q_{r}-\frac{k}{2}{\left(\tau q_{n}\right)}^{2}+(T-e_{r}q_{r})Q$$

In the Equation (1), $\left(p_{n}-c_{n}\right)$ represents the revenue of the OEM from selling unit new products, $(p_{n}-c_{n})q_{n}$ represents the total revenue of the OEM from selling new products, $\left(p_{r}-w\right)$ represents the revenue of the OEM from selling unit remanufactured products, $(p_{r}-w)q_{r}$ represents the total revenue earned by the OEM from selling remanufactured products, $e_{n}q_{n}$ represents the carbon emissions required by the OEM when the demand for new products is $q_{n}$, $e_{n}q_{n}-T$ represents the government giving the OEM a carbon emission credit T, the OEM needs to purchase additional carbon emission credits in order to meet the production needs, $(e_{n}q_{n}-T)Q$ represents the expenditure required by OEM to purchase carbon emission credits $e_{n}q_{n}-T$, so OEM’s revenue is the total revenue of new products and remanufactured products minus the expenditure of purchasing additional carbon emission credits.

In the Equation (2), $\left(w-c_{r}\right)$ represents the unit remanufactured products revenue to remanufacturer, $(w-c_{r})q_{r}$ represents the remanufactured products total revenue, $\frac{k}{2}{\left(\tau q_{n}\right)}^{2}$ represents the cost required for the remanufacturer to recycle waste products, $e_{r}q_{r}$ represents the carbon emissions required when the demand for remanufactured products is $q_{r}$, $T-e_{r}q_{r}$ represents that the carbon emission credit given to the remanufacturer is T, the carbon emission credit remaining after the remanufacturer produces the product, and $(T-e_{r}q_{r})Q$ represents the revenue obtained by the remanufacturer from selling the remaining $T-e_{r}q_{r}$ carbon emission credits. Therefore, the revenue of remanufacturers is equal to the total revenue of producing remanufactured products and selling residual carbon emission credits minus the cost of recycling waste products. 

### 3.2. Model Solving

In order to obtain the optimal solution, Lemma 1 is given.

**Lemma** **1.** *(i) In Equation (2),* $\pi_{r}$ *is a concave function about* τ *; (ii) in Equation (1),* $\pi_{n}$ *is a concave function about* $q_{n}$ *,* w *;*

According to Lemma 1, the optimal solution can be obtained, see Conclusion 1. See Appendix A for the Proof of Conclusion 1.

**Conclusion** **1.** *Under the carbon trade policy, the optimal solutions of the unit retail price of new products and remanufactured products, the sales volume of new products and remanufactured products, the revenue of OEMs and remanufacturers, the recycling rate of waste products, and the outsourcing cost of unit remanufactured product are as follows:*

$$p_{n}{}^{*}=\frac{1+c_{n}+e_{n}Q}{2}\;p_{r}{}^{*}=\delta \frac{\delta -\delta^{2}+k+k(c_{n}+e_{n}Q)+(1-\delta )(c_{r}+e_{r}Q)}{2(\delta -\delta^{2}+k)}\;q_{n}{}^{*}=\frac{\delta -\delta^{2}+k-(\delta +k)(c_{n}+e_{n}Q)+\delta (c_{r}+e_{r}Q)}{2(\delta -\delta^{2}+k)}\;q_{r}{}^{*}=\frac{\delta (c_{n}+e_{n}Q)-(c_{r}+e_{r}Q)}{2(\delta -\delta^{2}+k)}\;\pi_{n}^{*}=\frac{{\left[1-(c_{n}+e_{n}Q)\right]}^{2}}{4}+\frac{{\left[\delta (c_{n}+e_{n}Q)-(c_{r}+e_{r}Q)\right]}^{2}}{4(\delta -\delta^{2}+k)}+TQ\;\pi_{{}_{r}}^{*}=\frac{k}{8}{\left[\frac{\delta (c_{n}+e_{n}Q)-(c_{r}+e_{r}Q)}{\left(\delta -\delta^{2}+k\right)}\right]}^{2}+TQ\;\tau^{*}=\frac{\delta (c_{n}+e_{n}Q)-(c_{r}+e_{r}Q)}{\delta -\delta^{2}+k+\delta (c_{r}+e_{r}Q)-(\delta +k)(c_{n}+e_{n}Q)}\;w^{*}=\frac{k\delta (c_{n}+e_{n}Q)+(2\delta -2\delta^{2}+k)(c_{r}+e_{r}Q)}{2(\delta -\delta^{2}+k)}$$

Aiming at the above optimal solution, let Q=0 be the optimal solution when the government does not limit the carbon emission quota, i.e.,

$$p_{n}{}^{n*}=\frac{1+c_{n}}{2}\;p_{r}{}^{n*}=\delta \frac{\delta -\delta^{2}+k+kc_{n}+(1-\delta )c_{r}}{2(\delta -\delta^{2}+k)}\;q_{n}{}^{n*}=\frac{\delta -\delta^{2}+k-(\delta +k)c_{n}+\delta c_{r}}{2(\delta -\delta^{2}+k)}\;q_{r}{}^{n*}=\frac{\delta c_{n}-c_{r}}{2(\delta -\delta^{2}+k)}\;\pi_{n}^{n*}=\frac{{\left(1-c_{n}\right)}^{2}}{4}+\frac{{\left(\delta c_{n}-c_{r}\right)}^{2}}{4(\delta -\delta^{2}+k)}\;\pi_{{}_{r}}^{n*}=\frac{k}{8}{\left(\frac{\delta c_{n}-c_{r}}{\delta -\delta^{2}+k}\right)}^{2}\;\tau^{n*}=\frac{\delta c_{n}-c_{r}}{\delta -\delta^{2}+k+\delta c_{r}-(\delta +k)c_{n}}\;w^{n*}=\frac{k\delta c_{n}+(2\delta -2\delta^{2}+k)c_{r}}{2(\delta -\delta^{2}+k)}$$

### 3.3. Result Analysis

According to Conclusion 1, the impact of government carbon trade policy on outsourcing remanufacturing can be stated as follows:

**Conclusion** **2.** *The effects of carbon trade prices on unit outsourcing costs, unit retail prices, product sales volume, and the waste product recovery rate is as follows:*

*(i)* 
$\frac{\partial w^{*}}{\partial Q}=\frac{k\delta e_{n}+(2\delta -2\delta^{2}+k)e_{r}}{2(\delta -\delta^{2}+k)}>0$

*(ii)* 
$\frac{\partial p_{n}{}^{*}}{\partial Q}=\frac{e_{n}}{2}>0,\frac{\partial p_{r}{}^{*}}{\partial Q}=\delta \frac{ke_{n}+(1-\delta )e_{r}}{2(\delta -\delta^{2}+k)}>0$

*(iii)* 
$\frac{\partial q_{n}{}^{*}}{\partial Q}=\frac{-(\delta +k)e_{n}+\delta e_{r}}{2(\delta -\delta^{2}+k)}<0,$

*when* $\delta >\frac{e_{r}}{e_{n}}$ *,* $\frac{\partial q_{r}{}^{*}}{\partial Q}=\frac{\delta e_{n}-e_{r}}{2(\delta -\delta^{2}+k)}>0$ *, otherwise,* $\frac{\partial q_{r}{}^{*}}{\partial Q}<0$

*(iv)*  *When* $\delta >\frac{e_{r}}{e_{n}}$ *and* $\delta >\frac{c_{r}}{c_{n}}$ *,*
$$\frac{\partial \tau^{*}}{\partial Q}=\frac{\left(\delta -\delta^{2}+k\right)\left[\delta e_{n}-e_{r}-e_{n}(c_{r}+e_{r}Q)+e_{r}(c_{n}+e_{n}Q)\right]}{{\left[\delta -\delta^{2}+k+\delta (c_{r}+e_{r}Q)-(\delta +k)(c_{n}+e_{n}Q)\right]}^{2}}>0$$

See Appendix A for the Proof of Conclusion 2. Conclusion 2 shows that the carbon trade increases the outsourcing cost of unit remanufactured product paid by the OEM to remanufacturer, increases the retail price of unit new products and unit remanufactured products, and reduces the sales volume of new products. When $\delta >\frac{e_{r}}{e_{n}}$, the implementation of the carbon trade policy will increase the sales volume of remanufactured products. When δ meets the conditions of $\delta >\frac{e_{r}}{e_{n}}$ and $\delta >\frac{c_{r}}{c_{n}}$, carbon trade increases the recycling rate of waste products.

Since the carbon trade policy limits carbon emissions, OEMs have to purchase additional carbon emission credits. Therefore, OEMs increase the price of new products to cover the increased production costs and pass on the additional costs to consumers, causing a decline in product sales. In addition, to offset the negative impact of reduced sales of new products on revenue, OEMs increase the unit outsourcing costs of remanufactured products to promote the production and the sales of these products. Meanwhile, the production costs of remanufacturers have also increased, and remanufacturers thus demand higher outsourcing costs of unit remanufactured products. This leads to an increase in the sales cost of unit remanufactured products, and OEMs transfer the increased outsourcing cost to consumers indirectly, that is, carbon trade increases the sales price of unit new products and unit remanufactured products. When the ratio of the retail price of unit remanufactured product to unit new product is greater than the ratio of the unit carbon emissions of unit remanufactured product to unit new product. That is, consumers’ preference for remanufactured products is greater than the ratio of the environmental impact of remanufactured products and new products. It can be seen that as consumers’ preference for remanufactured products increases, carbon trade is conducive to setting up thresholds and increasing the sales volume of remanufactured products [26].

For remanufacturers, according to [56], the relative discount and the carbon emissions of unit product affect the sales volume of remanufactured products. The sales volume and the production cost of unit remanufactured products in turn affect the recycling rate of the waste products. Given the increased outsourcing fees, remanufacturers further increase production to make more profits, by enhancing the recycling rate of waste products. Different from [46], the reduction in production costs and carbon emissions benefit remanufacturers, increasing the incentives of remanufacturers for production and their demand for waste products. When the discount rate is higher than the ratio of the carbon emissions generated from remanufactured products to new products, a certain increase in the retail price of unit remanufactured products can increase the remanufacturer’s revenue. Therefore, remanufacturers will strive to reduce the carbon emissions and increase the retail price of remanufactured products. 

**Management Implication1:** OEMs can respond to the impact of carbon trade by changing product prices and production quantities. Remanufacturers need to consider discount rates, unit product costs, unit carbon emissions, and unit product outsourcing fees, in order to decide on production quantities and scrap recycling rates. As mentioned, the implementation of the carbon trade policy directly affects the manufacturing behavior of OEMs and remanufacturers. The government should also improve consumers’ environmental awareness and consumption preferences, and encourage remanufacturers to reduce carbon emission reduction costs through technological improvements.

Corollary 1 can be drawn from Conclusion 2, as follows:

**Corollary** **1.** *The effects of carbon trade on the unit retail price, sales volume, and unit outsourcing costs of the two products are as follows:*

*(i)* 
$p_{n}^{*}>p_{n}^{n*},p_{r}^{*}>p_{r}^{n*}$

*(ii)* 
$q_{n}^{*}<q_{n}^{n*}$

*when* $\delta >\frac{e_{r}}{e_{n}}$ *,* $q_{r}^{*}>q_{r}^{n*}$ *; otherwise,* $q_{r}^{*}\leq q_{r}^{n*}$

*(iii)* 
$w_{n}^{*}>w_{n}^{n*}$

Similar to [3], Corollary 1 shows that the implementation of carbon trade policy leads to an increase in the prices of the two products. Specifically, it directly increases the carbon emission costs and the overall production costs of new products. In turn, the sales volume of new products is likely to decline as OEMs usually increase sale prices of products to cover the increased costs. Meanwhile, carbon trade fees increase the outsourcing cost of unit remanufactured products and thus increase the overall cost of the remanufactured products sold by OEMs. As a result, remanufactured products will also see an increase in sales prices. Since carbon trade costs are directly amortized over new products, to maintain the sales volume of new and remanufactured products, OEMs need to adjust outsourcing fees based on the increasing level of carbon trade costs, and then weigh the benefits of the two products. It can be seen that the mechanism is very similar to the impact of the carbon tax on unit retail prices [54].

However, the carbon trade policy does not necessarily increase the sales volume of manufactured products under all conditions. It works only when the discount rate is greater than the ratio of carbon emissions from new products to remanufactured products. The sales volume of remanufactured products with the condition of the carbon trade policy is greater than that of remanufactured products without the condition. Being driven by the carbon trade policy, remanufacturers reduce the carbon emissions of unit remanufactured products by improving carbon reduction technologies, reducing carbon trade costs, and increasing the production scale.

**Conclusion** **3.** *The impact of carbon trade on the revenue of OEMs and remanufacturers are as follows:*

*(i)*  *when* $Q<\frac{\left(\delta -\delta^{2}+k)\left[e_{n}(1-c_{n})-2T\right]+(\delta e_{n}-e_{r})(c_{r}-\delta c_{n}\right)}{e_{n}^{2}(\delta -\delta^{2}+k)+{\left(\delta e_{n}-e_{r}\right)}^{2}}$ *,* $\frac{\partial \pi_{n}^{*}}{\partial Q}<0$ *,*
  *otherwise*$\frac{\partial \pi_{n}{}^{*}}{\partial Q}\geq 0$.
*(ii)*  *when* $\delta >\frac{e_{r}}{e_{n}}$ *and* $Q<\frac{\delta (c_{n}e_{r}+c_{r}e_{n}-\delta c_{n}e_{n})-c_{r}e_{r}-4\frac{T}{k}{\left(\delta -\delta^{2}+k\right)}^{2}}{\left(e_{n}-e_{r})(\delta e_{n}-e_{r}\right)}$ *,* $\frac{\partial \pi_{r}^{*}}{\partial Q}<0$ *,*
*otherwise* $\frac{\partial \pi_{r}^{*}}{\partial Q}\geq 0$ *;*
*when* $\delta <\frac{e_{r}}{e_{n}}$ *and* $Q<\frac{\delta (c_{n}e_{r}+c_{r}e_{n}-\delta c_{n}e_{n})-c_{r}e_{r}-4\frac{T}{k}{\left(\delta -\delta^{2}+k\right)}^{2}}{\left(e_{n}-e_{r})(\delta e_{n}-e_{r}\right)}$ *,* $\frac{\partial \pi_{r}^{*}}{\partial Q}>0$ *,*
  *otherwise*$\frac{\partial \pi_{r}^{*}}{\partial Q}\leq 0$.

See Appendix A for the Proof of Conclusion 3. Conclusion 3 shows that although carbon trade policy increases the unit retail price of the two products, it does not always enhance OEMs’ and remanufacturers’ revenue. OEMs’ revenue is proportional to the carbon trade prices only when the carbon trade price is above a certain threshold. When the carbon trade price is below a certain threshold, although the sales prices of new and remanufactured products increase, the sales volume of new products decreases significantly, which leads to a decline in the revenues created from new products. Meanwhile, the increase in revenues from remanufactured products is not enough to make up for the decrease in revenues from new products, and revenues from selling two products cannot make up for the increased costs caused by outsourcing fees and carbon trade fees, which in turn leads to a decrease in the overall revenues of an OEM. When the carbon trade price is higher than a certain threshold, remanufacturers reduce the carbon emissions associated with the production of remanufactured products, thereby selling more carbon emission credits to obtain revenues. According to Conclusion 2, the sales volume of remanufactured products will increase. In addition, when consumers realize that the carbon trade policy increases firms’ outsourcing costs and sales prices of products, they are willing to continue buying remanufactured products [57]. Therefore, a reasonable choice for OEMs is to sell more remanufactured products to make up for the loss of revenues caused by the decline in sales of new products.

When the ratio of carbon emissions of unit remanufactured products to that of new products is lower than the price ratio of the two products, the benefits to the remanufacturer are minimal depending on the carbon trade price. When the carbon trade price exceeds a certain point, the revenue of the remanufacturer increases as the carbon trade price increases. When carbon trade prices are low, however, it is not attractive for the remanufacturer to sell carbon emission credits to earn revenue. Moreover, when the carbon trade price reaches a certain threshold, the sales volume of remanufactured products may achieve the scale effect. Therefore, remanufacturers prefer to reduce the costs and obtain more benefits by improving emission reduction technologies. When the ratio of the carbon emissions of unit remanufactured products to that of new product is higher than the price ratio of the two products, the remanufacturer’s revenues reach the maximum at another certain point in the carbon trade price. When the carbon trade price is above this point, the remanufacturer’s revenues decrease as the carbon trade price increases. In this case, the carbon trade reduces the sales volume of remanufactured products. In the initial stage, with the increase in outsourcing fees and the retail price of unit remanufactured products, the revenues created from remanufactured products will increase. When the price of carbon trade rises to a certain level, the sales volume of remanufactured products and the recycling rate of waste products decline significantly, and the remanufacturer’s revenues therefore decrease. 

**Management Implication2:** As the carbon trade policy directly affects the revenue of the two manufacturers, the government should set a reasonable carbon trade price greater than a certain threshold, to increase the sales volume and promote the production motivation of the two types of manufacturers. Since carbon trade related costs cannot always be transferred from manufacturers to consumers through the market, the level of carbon trade prices is particularly important to both OEMs and remanufacturers. Therefore, the carbon trade price should not only include the direct carbon emissions generated from fossil fuel burning but also the indirect emissions from the use of electric heating. Otherwise, unreasonable carbon trade prices will hinder the low-carbon transformation of the two manufacturers [58].

In the condition of the carbon trade policy, the environmental impact of the OEM’s and remanufacturer’s production is $e=e_{n}q_{n}^{*}+e_{r}q_{r}^{*}$; when there is no carbon trade policy, the environmental impact of the OEM’s and remanufacturer’s production is $e^{n}=e_{n}q_{n}^{n*}+e_{r}q_{r}^{n*}$.

**Conclusion** **4.** *The impact of carbon trade on the environment is as follows:*$e<e^{n}$.

See Appendix A for the Proof of Conclusion 4. Conclusion 4 suggests that the adverse impact of the two manufacturers’ production activities on the environment is diminished by the carbon trade policy. In other words, the carbon trade policy helps mitigate the negative impact of the production activities of OEMs and remanufacturers on the environment. Conclusion 2 and Corollary 1 also show that the carbon trade policy shifts OEMs’ and remanufacturers’ production decisions and thus reduce their adverse influence on the environment, due to the reduced sales volume of new products. In addition, the carbon trade policy directly increases the production costs of OEMs, forcing OEMs to reduce carbon emissions through technological innovation.

**Management Implication3:** The government needs to promote the efficiency of the carbon trade market, encourage OEMs to carry out low-carbon production activities through a wide range of means, such as providing subsidies based on carbon emission levels. Meanwhile, to enhance public support for low-carbon remanufactured products, the government could launch campaigns to educate the public and increase consumers’ environmental awareness.

**Conclusion** **5.** *The impact of carbon trade on consumer surplus is as follows:*

*When*$Q>\frac{2e_{n}(1-c_{n}){\left(\delta -\delta^{2}+k\right)}^{2}+2(\delta -\delta^{2})\left[\delta (c_{n}e_{r}+c_{r}e_{n})-c_{n}e_{n}\delta^{2}-c_{r}e_{r}\right]}{e_{n}^{2}{\left(\delta -\delta^{2}+k\right)}^{2}+(\delta -\delta^{2}){\left(\delta e_{n}-e_{r}\right)}^{2}}$*,*$S>S^{n}$*, otherwise*$S\leq S^{n}$.

See Appendix A for the Proof of Conclusion 5. Conclusion 5 shows that carbon trade does not always increase consumer surplus. Carbon trading price above a certain threshold is a necessary condition for increasing consumer surplus.

**Management Implication4:** The government can help increase consumer surplus by setting a reasonable carbon trade price, thereby protecting the interests of consumers. In addition, under the role of an effective market, the carbon trade policy can enable consumers to gain more benefits in market transactions, thereby encouraging consumers to purchase remanufactured products continuously.

## 4. Numerical Analysis

To further analyze the impact of the carbon trade policy under the condition of outsourcing remanufacturing on both new and remanufactured products, environmental benefits, and consumer surplus, this article conducts a numerical analysis by taking the case of a medium-sized used engine remanufacturing firm in China. According to [56], compared with a new engine, a unit remanufactured engine can save the production cost by 50% and reduce the environmental impact by 60%. Therefore, the production cost and environmental impact of unit new products and unit remanufactured products can be set as: $c_{n}=0.2\;,c_{r}=0.1,\;e_{n}=1,\;e_{r}=0.4$, According to [59], set k=1.1, δ=0.6 ,T=2.

### 4.1. The Impact of Q and δ on the Unit Retail Prices of Two Products

**Figure 1:**
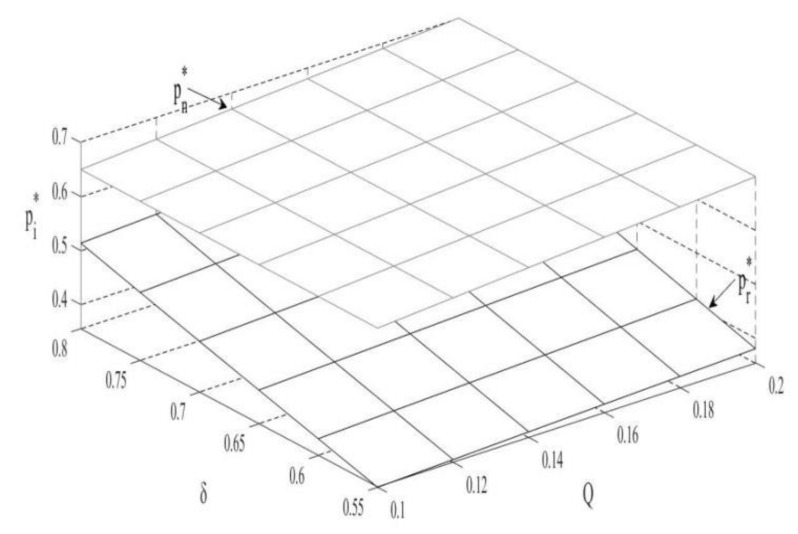
The impact of Q and δ on the unit retail prices.

According to Figure 1, the unit retail price of the two products is proportional to the carbon trade prices. The main reason is that the carbon trade policy increases the production costs of new products, and OEMs will pass on such costs to consumers by raising the price. At the same time, the carbon trade policy also increases outsourcing costs and remanufactured product costs, which in turn increases the price of remanufactured products.

**Corollary** **2.** *The impact of consumer preferences on the unit retail prices of two products:*

*(i)* 
$\frac{\partial p_{n}{}^{*}}{\partial \delta }=0,\frac{\partial p_{r}{}^{*}}{\partial \delta }>0$

Similar to [54], the unit retail price of new products is theoretically not affected by consumer preference. As the public favors remanufactured products, however, the OEMs will see decreased sales of new products and potential profit loss under the carbon trade policy. To offset such potential loss, the OEMs no longer increase the retail prices of new products. In contrast, the unit retail price of remanufactured products is proportional to consumer preferences, which is conducive to enhancing the market competitiveness of remanufactured products. In turn, remanufacturers will increase the retail prices of remanufactured products to gain more profits.

### 4.2. The Impact of Q and δ on the Sales Volume of Two Products

**Figure 2:**
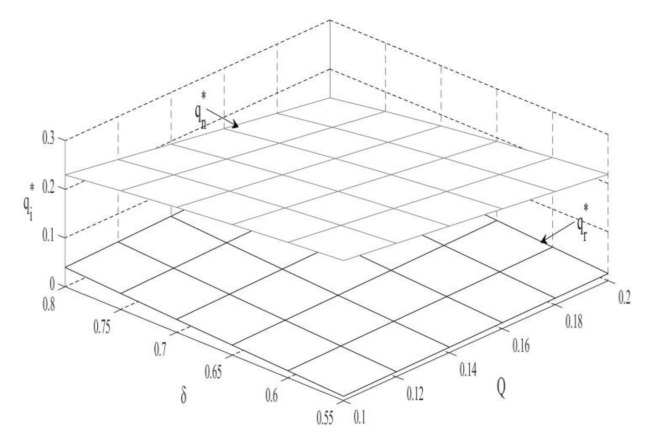
The impact of Q and δ on the sales volume.

According to Figure 2, the sales volume of new products is inversely proportional to the carbon trade price, and the sales volume of remanufactured products is directly proportional to the carbon trade price. As the price of carbon trade increases, the production cost and sales price of new products will increase substantially, which will reduce consumer purchases and consumers will switch to remanufactured products. In addition, rising carbon trade prices attract remanufacturers to reduce carbon emissions and expand production to obtain greater profits. For OEMs, the increase in sales volume of remanufactured products can offset some of the losses caused by the decline in sales volume of new products. Therefore, the increase in carbon trade prices will increase the sales volume of remanufactured products and reduce the sales volume of new products.

**Corollary** **3.** *The impact of consumer preferences on the sales of two products:*

*(i)* 
$\frac{\partial q_{n}{}^{*}}{\partial \delta }<0,\frac{\partial q_{r}{}^{*}}{\partial \delta }>0$

The sales volume of new products is inversely proportional to consumer preferences, and the sales volume of remanufactured products is directly proportional to consumer preferences. This is due to that the market prospects for remanufactured products have greatly improved as consumer preferences increase, so OEMs will increase the market share of remanufactured products and squeeze the market share and sales volume of new products [57].

### 4.3. The Impact of Q and δ on the Recovery Rate of Waste Products 

**Figure 3:**
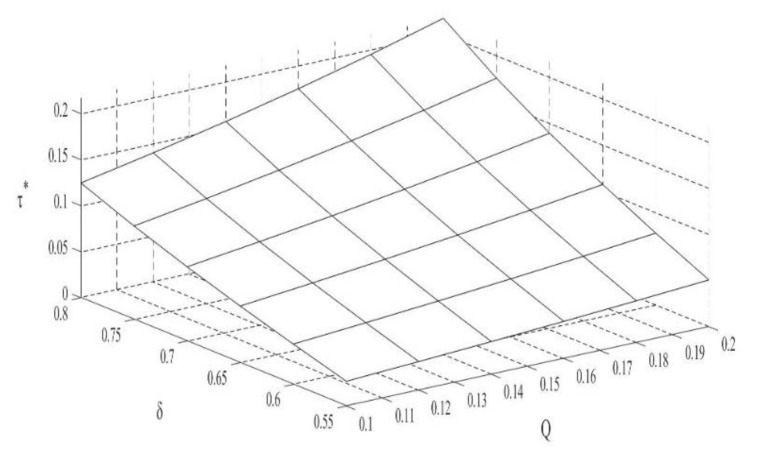
The impact of Q and δ on the recovery rate of waste products.

According to Figure 3, the recycling rate of waste products is directly proportional to the carbon trade price. This is because implementation of the carbon trade policy will increase the sales volume of remanufactured products. Therefore, to steadily increase the scale of their production, remanufacturers will increase the recovery of waste products.

**Corollary** **4.** *The influence of consumer preferences on the recycling rate of waste products:*

*(i)* 
$\frac{\partial \tau^{*}}{\partial \delta }>0$

According to Corollary 3, the increase in consumer preference for remanufactured products will increase the sales volume of remanufactured products. Therefore, remanufacturers will increase the recycling volume of waste products to meet market demand and obtain greater benefits.

### 4.4. The Impact of Q and δ on the Revenue of OEMs and Remanufacturers 

**Figure 4:**
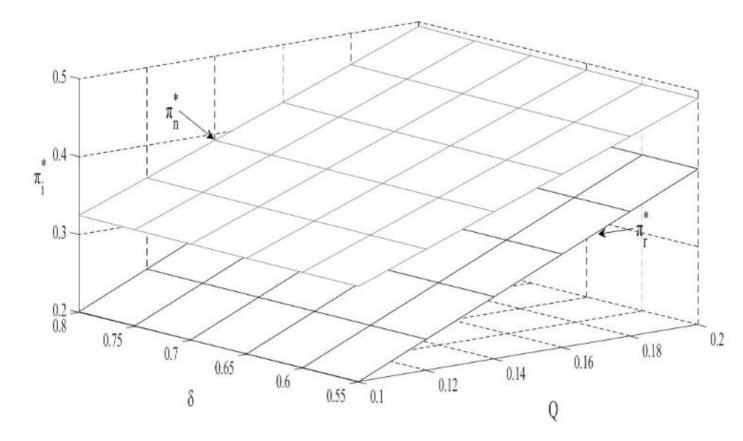
The impact of Q and δ on the revenue.

According to Figure 4, the revenues of OEMs and remanufacturers are directly proportional to carbon trade prices. With an increase in carbon trade prices, the unit retail price and sales volume of remanufactured products will increase, leading to an increase of the remanufacturers’ revenues. For OEMs, the substantial increase in revenue from remanufactured products can offset some of the losses caused by the reduction in sales of new products. In addition, the production costs of remanufactured products are relatively lower, which leads to an increase in the overall revenues of the OEM.

**Corollary** **5.** *The impact of consumer preferences on revenue:*

*(i)* 
$\frac{\partial \pi_{n}^{*}}{\partial \delta }>0,\frac{\partial \pi_{r}^{*}}{\partial \delta }>0$

The increase in consumer preferences for remanufactured products is conducive to expand remanufacturing market share and increase the production scale of remanufacturers. As a result, outsourcing fees and the benefits generated from the carbon trade will also increase, further enhancing the overall benefits of remanufacturers. 

## 5. Conclusions

To examine the impacts of carbon trade policy on outsourcing remanufacturing, this article constructs a game model between an OEM and a remanufacturer under the conditions of carbon trade policy. It compares and analyzes different impacts of carbon trade on the profits and behavioral decisions of both parties. The main conclusions are as follows.

(1) The carbon trade price is proportional to outsourcing costs, retail prices of new products, and retail prices of remanufactured products. The carbon trade price is inversely proportional to the sales volume of new products. Only when the carbon trade price is above a certain threshold can carbon trade increase the revenues of the OEM and the remanufacturer.
(2) In addition to carbon trade prices, consumer preferences and unit carbon emissions are also factors that the manufacturers need to consider in decision-making. The increase in consumer preferences for remanufactured products and the reduction of carbon emissions associated with remanufactured products are conducive to increasing the sales volume of remanufactured products, the recycling rate of waste products, and the revenues. Therefore, when making decisions, manufacturers should consider various factors and constantly seek new technologies to achieve carbon emissions reduction and maximize profits.
(3) The carbon trade policy reduces the adverse effects of manufacturing activities on the environment, but they do not always increase consumer surplus. Only when the carbon trade price is above a certain threshold can carbon trade increase consumer surplus. Therefore, the government should set an appropriate carbon trade price to guide resource allocation. Meanwhile, the government needs to launch a propaganda subsidy policy to improve the visibility and penetration rate of remanufactured products, and encourage manufacturers to voluntarily reduce emissions, thus supporting the rapid growth of the remanufacturing industry.

Based on these findings, this research field can be further explored in the future. First, carbon trade is the trading of intangible assets, and it thus faces high transaction costs in all aspects of activities of verification, grading, and trading. As the goal of carbon neutrality is approaching, the prospects for the development of carbon trade remain unclear. At the same time, China’s policy practice has preliminarily shown that the separate implementation of carbon trade policy and carbon tax policy have a higher implementation cost. The combined use of the two policies suggests a more feasible government strategy in the future. Therefore, the impacts of the mixed policy of carbon trade and carbon tax on outsourcing remanufacturing deserve more attention. Second, studies on the current carbon trade pricing mechanism are not sound, and carbon trade pricing is still a combination of paid-free carbon emissions reduction. The further analysis of the impacts of all-paid carbon trade pricing mechanisms on outsourcing remanufacturing is meaningful, given the fact that the cutoff for peak carbon is approaching. Third, the current mainstream carbon trade policy worldwide is mainly to establish a regional carbon trade market. As the internationalization and industrialization of carbon trade deepen in the future, further research on the impacts of the carbon trade market mechanism based on relatively complete and customized quota allocation on outsourcing remanufacturing is needed.

## Figures and Tables

**Table 1:** Definition of symbols.

Symbol	Definition
n,r	OEM, remanufacturer
Superscript n	The government does not carry out carbon trade policy;
$q_{n},q_{r}$	Sales volume of new and remanufactured products;
$p_{n},p_{r}$	Unit retail prices of new and remanufactured products;
$c_{n},c_{r}$	Unit production cost of new and remanufactured products (in reality it is known that $c_{n}>c_{r}$);
T	The carbon emission credits given to manufacturers by the government;
$e_{n},e_{r}$	The carbon emissions of unit new product and unit remanufactured product (that is, the impact of unit new product and unit remanufactured product on the environment, it is that in reality $e_{n}>e_{r}$);
$e_{}$	The total carbon emissions of new products and remanufactured products, that is, the total impact of the two manufacturers’ production on the environment;
Q	Unit carbon emissions trade price;
τ	The ratio of the number of waste products recycled by remanufacturers to the sales volume of new products (that is, the recycling rate of waste products);
δ	The ratio of the retail price of a unit remanufactured product to the retail price of a new product, which indicates the consumer’s preference for remanufactured products (in reality, it is known that 0≤δ≤1);
w	When the production unit remanufactures the product, the outsourcing fee paid by OEM to the remanufacturer;
$\pi_{n},\pi_{r}$	Revenue of OEMs and remanufacturers.

## Data Availability

The data come from a medium-size used engine remanufacturing firm in China, Jinan Fuqiang Company.

## Appendix A

**Proof.** Proof of Conclusion 1. Substituting $p_{Vr}=\delta (1-q_{Vn}-q_{Vr}),q_{Vr}=\tau_{V}q_{V1}$ into Equation (2), we can rewrite $\pi_{Vr}\;$as follows:

(A1) $$\pi_{Vr}=(1-q_{Vn}-\delta \tau_{Vr}q_{V1}-z_{V}+v)\tau_{Vr}q_{V1}-\frac{k}{2}{\left(\tau_{Vr}q_{V1}\right)}^{2}$$

The first-order and second-order derivatives of $\pi_{Vr}$ concerning $\tau_{V}$ are as follows:

(A2) $$\frac{\partial \pi_{Vr}}{\partial \tau_{Vr}}=(1-q_{Vn}-z_{V}+v)q_{V1}-2\delta \tau_{Vr}q_{V1}{}^{2}-k\tau_{Vr}q_{V1}{}^{2}$$

(A3) $$\frac{\partial^{2}\pi_{Vr}}{\partial \tau_{Vr}{}^{2}}=-2\delta q_{V1}{}^{2}-kq_{V1}{}^{2}<0$$

Because the second-order sufficient condition in Equation (A3) is negative, the revenue function $\pi_{Vr}\;$ in Equation (2) is concave in $\tau_{V}$. Let Equation (A2) equal 0 and by solving them simultaneously, we obtain:

(A4) $$\tau_{V}^{*}=\frac{1-q_{Vn}-z_{V}+v}{(2\delta +k)q_{V1}}$$

Substituting $p_{V1}=1-q_{V1},\;p_{Vn}=1-q_{Vn}-\delta q_{Vr},\;p_{Vr}=\delta (1-q_{Vn}-q_{Vr}),\;q_{Vr}=\tau_{V}^{*}q_{V1}$ into Equation (1), the revenue function of $\pi_{Vn}$ can be rewritten as follows:

(A5) $$\pi_{Vn}=(1-q_{V1}-c_{n})q_{V1}+(1-q_{Vn}-\delta \frac{1-q_{Vn}-z_{V}+v}{2\delta +k}-c_{n})q_{Vn}+z_{V}\frac{1-q_{Vn}-z_{V}+v}{2\delta +k}$$

The first-order and second-order derivatives of $\pi_{Vn}\;$ in Equation (A5) concerning *q_V_*_1_, *q_Vn_*, and *z_V_* are as follows:

$$\frac{\partial \pi_{Vn}}{\partial q_{V1}}=1-2q_{V1}-c_{n}$$

$$\frac{\partial \pi_{Vn}}{\partial q_{Vn}}=\frac{(2\delta +k)(1-c_{n})-\delta (\delta +v-c_{r})-2(2\delta +k-\delta^{2})q_{Vn}}{2\delta +k}$$

$$\frac{\partial \pi_{Vn}}{\partial z}=\frac{\delta +v-c_{r}-2z}{2\delta +k}$$

$$\frac{\partial^{2}\pi_{Vn}}{\partial q_{V1}{}^{2}}=-2,\;\frac{\partial^{2}\pi_{Vn}}{\partial q_{Vn}\partial q_{V1}}=\frac{\partial^{2}\pi_{Vn}}{\partial \tau_{V}\partial q_{V1}}=0$$

$$\frac{\partial^{2}\pi_{Vn}}{\partial z^{2}}=-\frac{2}{2\delta +k},\;\frac{\partial^{2}\pi_{Vn}}{\partial q_{Vn}\partial z}=\frac{\partial^{2}\pi_{Vn}}{\partial q_{V1}\partial z}=0$$

$$\frac{\partial^{2}\pi_{Vn}}{\partial q_{Vn}{}^{2}}=-2\frac{2\delta +k-\delta^{2}}{2\delta +k},\;\frac{\partial^{2}\pi_{Vn}}{\partial q_{V1}\partial q_{Vn}}=\frac{\partial^{2}\pi_{Vn}}{\partial \tau_{V}\partial q_{Vn}}=0$$

Moreover, the Hessian matrix of $\pi_{Vn}$ in Equation (A5) on *q_V_*_1_, *q_Vn_*, and *z_V_* is as follows:

$$H=\left[\begin{array}{ccc} -2 & 0 & 0\\ 0 & -2\frac{2\delta +k-\delta^{2}}{2\delta +k} & 0\\ 0 & 0 & -\frac{2}{2\delta +k} \end{array}\right]$$

The first-principal, second-principal, and third-principal minors of the Hessian matrix *H* are calculated as follows:

$${\left|H\right|}_{1}=\left|-2\right|=2>0,$$

$$\;{\left|H\right|}_{2}=\left|\begin{array}{cc} -2 & \\ & -2\frac{2\delta +k-\delta^{2}}{2\delta +k} \end{array}\right|=4\frac{2\delta +k-\delta^{2}}{2\delta +k}>0$$

$$H_{3}=\left|\begin{array}{ccc} -2 & 0 & 0\\ 0 & -2\frac{2\delta +k-\delta^{2}}{2\delta +k} & 0\\ 0 & 0 & -\frac{2}{2\delta +k} \end{array}\right|=-8\frac{2\delta +k-\delta^{2}}{{\left(2\delta +k\right)}^{2}}<0$$

Therefore, Equation (A5) is concave to *q_V_*_1_, *q_Vn_*, and *z_V_*,. That is, Equation (1) is concave to *q_V_*_1_, *q_Vn_*, and *z_V_*. Conclusion 1b could be proven similarly. The proof of Conclusion 1 is completed. □

**Proof.** Proof of Conclusion 2. We can see from Conclusion 1:

$$\frac{\partial p_{V1}{}^{*}}{\partial v}=\frac{\partial p_{Vn}{}^{*}}{\partial v}=\frac{\partial \frac{1+c_{n}}{2}}{\partial v}=0,\;\frac{\partial p_{Vr}{}^{*}}{\partial v}=-\frac{\delta (1-\delta )}{2(2\delta +k-\delta^{2})}<0,$$

(a) is proven. (b), (c) and (d) can be proven similarly. The proof of Conclusion 2 is completed. □

**Proof.** Proof of Conclusion 3. We can see from Conclusion 1:

$$\frac{\partial z_{V}{}^{*}}{\partial v}=\frac{1}{2}>0,\;\frac{\partial z_{S}{}^{*}}{\partial s}=0;$$

$$\frac{\partial \tau_{V}{}^{*}}{\partial v}=\frac{1}{\left(2\delta +k-\delta^{2})(1-c_{n}\right)}>0,\;\frac{\partial \tau_{S}{}^{*}}{\partial s}=\frac{\delta (1+c_{n})-c_{r}}{(2\delta +k-\delta^{2}){\left(1-c_{n}-s\right)}^{2}}>0$$

The proof of Conclusion 3 is completed. □

**Proof.** Proof of Conclusion 4. We can see from Conclusion 1:

$$E_{N}=e_{n}[\frac{1-c_{n}}{2}+\frac{1}{2}-\frac{(k+2\delta )c_{n}-\delta c_{r}}{2(2\delta +k-\delta^{2})}]+e_{r}\frac{v-c_{r}+\delta c_{n}}{2(2\delta +k-\delta^{2})},$$

$$E_{V}=E_{N}+v\frac{e_{r}-\delta e_{n}}{2(2\delta +k-\delta^{2})},$$

$$E_{S}=E_{N}+s\frac{\delta e_{r}-e_{n}(4\delta +2k-\delta^{2})}{2(2\delta +k-\delta^{2})},$$

When $\frac{e_{r}}{e_{n}}>\delta$, $\frac{e_{r}-\delta e_{n}}{2(2\delta +k-\delta^{2})}>0$, that is,

$$E_{V}-E_{N}=v\frac{e_{r}-\delta e_{n}}{2(2\delta +k-\delta^{2})}>0.$$

Thus, (a) is proven. (b) and (c) can be proven similarly. The proof of Conclusion 4 is completed. □

**Proof.** Proof of Conclusion 5. We can see from Conclusion 1:

$$S_{N}=\frac{{\left(\frac{1-c_{n}}{2}\right)}^{2}+\delta (1-\delta ){\left[\frac{\delta c_{n}-c_{r}}{2(2\delta +k-\delta^{2})}\right]}^{2}}{2},$$

$$S_{V}=\frac{{\left(\frac{1-c_{n}}{2}\right)}^{2}+\delta (1-\delta ){\left[\frac{v+\delta c_{n}-c_{r}}{2(2\delta +k-\delta^{2})}\right]}^{2}}{2},$$

$$S_{V}-S_{N}=\delta (1-\delta )\frac{{\left[\frac{v+\delta c_{n}-c_{r}}{2(2\delta +k-\delta^{2})}\right]}^{2}-{\left[\frac{\delta c_{n}-c_{r}}{2(2\delta +k-\delta^{2})}\right]}^{2}}{2},$$

$$\frac{v+\delta c_{n}-c_{r}}{2(2\delta +k-\delta^{2})}-\frac{\delta c_{n}-c_{r}}{2(2\delta +k-\delta^{2})}=\frac{v}{2(2\delta +k-\delta^{2})}>0,$$

that is, $S_{V}>S_{N}$. Similar proof $S_{N}>S_{S}$. The proof of Conclusion 5 is completed. □

## References

[1] Jafar H., Maryam G. (2018). A revenue sharing contract for reverse supply chain coordination under stochastic quality of returned products and uncertain remanufacturing capacity. J. Clean. Prod..

[2] Shuai C., Chen X., Wu Y., Tan Y., Zhang Y., Shen L. (2018). Identifying the key impact factors of carbon emission in China: Results from a largely expanded pool of potential impact factors. J. Clean. Prod..

[3] Chai Q., Xiao Z., Lai K.H., Zhou G. (2018). Can carbon cap and trade mechanism be beneficial for remanufacturing?. Int. J. Prod. Econ..

[4] Hu X., Yang X.J., Sun J., Zhang Y.L. (2020). Carbon Tax or Cap-and-Trade: Which is More Viable for Chinese Remanufacturing Industry?. J. Clean. Prod..

[5] Flachsland C., Marschinski R., Edenhofer O. (2009). To link or not to link: Benefits and disadvantages of linking cap-and-trade systems. Clim. Policy.

[6] Knight E.R.W. (2011). The economic geography of European carbon market trading. J. Econ. Geogr..

[7] Nabernegg S., Bednar-Friedl B., Wagner F., Schinko T., Cofala J., Clement Y.M. (2017). The Deployment of Low Carbon Technologies in Energy Intensive Industries: A Macroeconomic Analysis for Europe, China and India. Energies.

[8] Jevnaker T., Wettestad J. (2017). Ratcheting Up Carbon Trade: The Politics of Reforming EU Emissions Trading. Glob. Environ. Polit..

[9] Naegele H., Zaklan A. (2019). Does the EU ETS cause carbon leakage in European manufacturing?. J. Environ. Econ. Manag..

[10] Tang R.H., Guo W., Oudenes M., Li P., Wang J., Tang J., Wang L., Wang H.J. (2018). Key challenges for the establishment of the monitoring, reporting and verification (MRV) system in China’s national carbon emissions trade market. Clim. Policy.

[11] Chang X.Y., Li Y.P., Zhao Y.B., Liu W.J., Wu J. (2017). Effects of carbon permits allocation methods on remanufactured production decisions. J. Clean. Prod..

[12] Tang B.J., Ji C.J., Hu Y.J., Tan J.X., Wang X.Y. (2020). Optimal carbon allowance price in China’s carbon emission trade system: Perspective from the multi-sectoral marginal abatement cost. J. Clean. Prod..

[13] Weitzman M.L. (2019). For international cap-and-trade in carbon permits, price stabilization introduces secondary free-rider-type problems. Environ. Resour. Econ..

[14] Bruninx K., Ovaere M., Delarue E. (2020). The long-term impact of the market stability reserve on the EU emission trade system. Energy Econ..

[15] Savaskan R.C., Bhattacharya S., Van Wassenhove L.N. (2004). Closed-Loop Supply Chain Models with Product Remanufacturing. Manag. Sci..

[16] Li J., González M., Zhu Y. (2009). A hybrid simulation optimization method for production planning of dedicated remanufacturing. Int. J. Prod. Econ..

[17] Faraca G., Edjabou V.M., Boldrin A., Astrup T. (2019). Combustible waste collected at Danish recycling centres: Characterisation, recycling potentials and contribution to environmental savings. Waste Manag..

[18] Xu L., Wang C. (2018). Sustainable manufacturing in a closed-loop supply chain considering emission reduction and remanufacturing. Resour. Conserv. Recycl..

[19] Zhou J., Deng Q.W., Li T. (2018). Optimal acquisition and remanufacturing policies considering the effect of quality uncertainty on carbon emissions. J. Clean. Prod..

[20] Xia X.Q., Zhang C.X. (2019). The impact of authorized remanufacturing on sustainable remanufacturing. Processes.

[21] Oersdemir A., Kemahlioglu-Ziya E., Parlaktuerk A.K. (2014). Competitive Quality Choice and Remanufacturing. Prod. Oper. Manag..

[22] Zhu X., Ren M., Chu W., Chiong R. (2019). Remanufacturing subsidy or carbon regulation? An alternative toward sustainable production. J. Clean. Prod..

[23] Long X.F., Ge J.L., Shu T., Liu Y. (2019). Analysis for recycling and remanufacturing strategies in a supply chain considering consumers’ heterogeneous WTP. Resour. Conserv. Recycl..

[24] Zou Z.B., Wang J.J., Deng G.S., Chen H.Z. (2016). Third-party remanufacturing mode selection: Outsourcing or authorization?. Transp. Res. Pt. e-Logist. Transp. Rev..

[25] Zhang Y.M., Chen W.D., Mi Y. (2020). Third-party remanufacturing mode selection for competitive closed-loop supply chain based on evolutionary game theory. J. Clean. Prod..

[26] Yan W., Li H.Y., Chai J.W., Qian Z.F., Chen H. (2018). Owning or Outsourcing? Strategic Choice on Take-Back Operations for Third-Party Remanufacturing. Sustainability.

[27] Qian Z.F., Chai J.W., Li H.Y., Yan W., Chen H. (2020). Should OEMs Outsource Remanufacturing to Retailers?. Asia Pac. J. Oper. Res..

[28] Mashhadi A.R., Behdad S. (2017). Optimal sorting policies in remanufacturing systems: Application of product life-cycle data in quality grading and end-of-use recovery. J. Manuf. Syst..

[29] Mitra S., Webster S. (2008). Competition in remanufacturing and the effects of government subsidies. Int. J. Prod. Econ..

[30] Li J., Du W., Yang F., Hua G. (2014). The carbon subsidy analysis in remanufacturing closed-loop supply chain. Sustainability.

[31] Helveston J.P., Liu Y., Feit E.M., Fuchs E., Klampfl E., Michalek J.J. (2015). Will subsidies drive electric vehicle adoption? Measuring consumer preferences in the US and China. Transp. Res. Pt. A-Policy Pract..

[32] Lu Z., Shao S. (2016). Impacts of government subsidies on pricing and performance level choice in energy performance contracting: A two-step optimal decision model. Appl. Energy.

[33] He P., He Y., Xu H. (2019). Channel structure and pricing in a dual-channel closed-loop supply chain with government subsidy. Int. J. Prod. Econ..

[34] Cao J., Chen X.H., Zhang X.M., Gao Y.C., Zhang X.P., Kumar S. (2020). Overview of remanufacturing industry in China: Government policies, enterprise, and public awareness. J. Clean. Prod..

[35] Li L., Guo S., Cai H., Wang J., Zhang J., Ni Y. (2020). Can China’s BEV market sustain without government subsidies?: An explanation using cues utilization theory. J. Clean. Prod..

[36] Yang M., Zhang L., Dong W. (2020). Economic benefit analysis of charging models based on differential electric vehicle charging infrastructure subsidy policy in China. Sustain. Cities Soc..

[37] Gu X.Y., Zhou L., Huang H.F., Shi X.T., Ieromonachou P. (2021). Electric vehicle battery secondary use under government subsidy: A closed-loop supply chain perspective. Int. J. Prod. Econ..

[38] Qiao H.K., Su Q. (2021). Impact of government subsidy on the remanufacturing industry. Waste Manag..

[39] Esenduran G., Kemahlıoğlu-Ziya E., Swaminathan J.M. (2017). Impact of take-back regulation on the remanufacturing industry. Prod. Oper. Manag..

[40] Huang Y., Wang Z. (2017). Closed-loop supply chain models with product take-back and hybrid remanufacturing under technology licensing. J. Clean. Prod..

[41] Li B.Y., Wang Y., Wang Z. (2021). Managing a closed-loop supply chain with take-back legislation and consumer preference for green design. J. Clean. Prod..

[42] Wang C., Wang W., Huang R. (2017). Supply chain enterprise operations and government carbon tax decisions considering carbon emissions. J. Clean. Prod..

[43] Alegoz M., Kaya O., Bayindir Z.P. (2021). A comparison of pure manufacturing and hybrid manufacturing-remanufacturing systems under carbon tax policy. Eur. J. Oper. Res..

[44] Dou G.W., Guo H.N., Zhang Q.Y., Li X.D. (2019). A two-period carbon tax regulation for manufacturing and remanufactured production planning. Comput. Ind. Eng..

[45] Saxena L.K., Jain P.K., Sharma A.K. (2018). Tactical supply chain planning for tyre remanufacturing considering carbon tax policy. Int. J. Adv. Manuf. Tech..

[46] Haites E. (2018). Carbon taxes and greenhouse gas emissions trading systems: What have we learned?. Clim. Policy.

[47] Chen Y.Y., Li B.Y., Zhang G.Q., Bai Q.G. (2020). Quantity and collection decisions of the remanufacturing enterprise under both the take-back and carbon emission capacity regulations. Transp. Res. E.

[48] Taleizadeh A.A., Noori-daryan M., Govindan K. (2016). Pricing and ordering decisions of two competing supply chains with different composite policies: A Stackelberg game theoretic approach. Int. J. Prod. Res..

[49] Bi H.M., Xiao H., Sun K.J. (2019). The impact of carbon market and carbon tax on green growth pathway in China: A Dynamic CGE Model Approach. Emerg. Mark. Financ. Trade.

[50] Shu T., Huang C.F., Chen S., Wang S.Y., Lai K.K. (2018). Trade-old-for-remanufactured closed-loop supply chains with carbon tax and government subsidies. Sustainability.

[51] Bian J., Zhao X. (2020). Tax or subsidy? An analysis of environmental policies in supply chains with retail competition. Eur. J. Oper. Res..

[52] Cao K., He P., Liu Z. (2020). Production and pricing decisions in a dual-channel supply chain under remanufacturing subsidy policy and carbon tax policy. J. Oper. Res. Soc..

[53] Zhang Y., Hong Z., Chen Z., Glock C.H. (2020). Tax or subsidy? Design and selection of regulatory policies for remanufacturing. Eur. J. Oper. Res..

[54] Li B., Geng Y., Xia X.X., Qiao D., Wang H. (2021). Comparatively analyzing the impact of government subsidy and carbon tax policy on authorized remanufacturing. Int. J. Environ. Res. Public Health.

[55] Ding J.F., Chen W.D., Wang W.B. (2020). Production and carbon emission reduction decisions for remanufacturing firms under carbon tax and take-back legislation. Comput. Ind. Eng..

[56] Zhao S.L., Zhu Q.H. (2017). Remanufacturing supply chain coordination under the stochastic remanufacturability rate and the random demand. Ann. Oper. Res..

[57] Hu S., Dai Y., Ma Z.J., Ye Y.S. (2016). Designing contracts for a reverse supply chain with strategic recycling behavior of consumers. Int. J. Prod. Econ..

[58] Govindan K., Popiuc M.N. (2014). Reverse supply chain coordination by revenue sharing contract: A case for the personal computers industry. Eur. J. Oper. Res..

[59] Yi P.X., Huang M., Guo L.J., Shi T.L. (2016). Dual recycling channel decision in retailer oriented closed-loop supply chain for construction machinery remanufacturing. J. Clean. Prod..

